# Mindfulness in Family Caregivers of Persons with Dementia: Systematic Review and Meta-Analysis

**DOI:** 10.3390/healthcare8030193

**Published:** 2020-06-30

**Authors:** María del Mar Molero Jurado, María del Carmen Pérez-Fuentes, Ana Belén Barragán Martín, José Gabriel Soriano Sánchez, Nieves Fátima Oropesa Ruiz, Maria Sisto, José Jesús Gázquez Linares

**Affiliations:** 1 Department of Psychology, Faculty of Psychology, University of Almería, 04120 Almería, Spain; mmj130@ual.es (M.d.M.M.J.); abm410@ual.es (A.B.B.M.); jss955@ual.es (J.G.S.S.); foropesa@ual.es (N.F.O.R.); ms168@ual.es (M.S.); 2 Department of Psychology, Faculty of Psychology, Universidad Politécnica y Artística del Paraguay, Asunción 1628, Paraguay; 3 Department of Psychology, Universidad Autónoma de Chile, Santiago 4780000, Chile; jlinares@ual.es

**Keywords:** dementia, caregivers, meta-analysis, mindfulness, systematic review

## Abstract

Social and healthcare problems associated with dementia not only affect those who suffer from this disease, but their caregivers as well. The purpose of this study was to analyze the efficacy of mindfulness intervention on psychological variables of caregivers of persons with dementia. A search for scientific articles published from 2000 to 2019 in the PubMed, Web of Science and PsycINFO databases found a total of 282 articles. After screening with preestablished inclusion criteria, ten studies with participation of 161 caregivers remained for the meta-analysis. The results were significant in favor of mindfulness intervention for the variables studied with a standardized difference of mean of 0.71 at a 95% CI, 0.71 (0.52, 0.89); *p* ≤ 0.00001. Heterogeneity of the studies included was moderate (*I*^2^ = 40%). The main conclusion suggested by empirical evidence was that mindfulness intervention seems to be effective for the variables analyzed. However, continued in-depth study of this subject is recommended.

## 1. Introduction

Research on mindfulness-based intervention (MBI) has grown significantly, as shown, for example, by recent studies in the clinical environment with fibromyalgia patients by Amutio et al. [1] or in a normalized population with a sample of public employees by Macías, Valero-Aguayo, Bond, and Blanca [2]. This progress in research has been due partly to the findings of studies which agree that practicing mindfulness is psychologically beneficial as it reduces symptoms of anxiety, stress, insomnia and depression [3,4,5,6,7,8] and promotes wellbeing by influencing mood and thinking [9]. 

MBI are “third generation” or “third-wave behavior” therapies. They are based on acceptance, receptiveness of how we feel, establishing a balanced, healthy coexistence with our own feelings, sentiments and thoughts, instead of attempting change from the beginning. One of its most relevant focuses is found in the theoretical proposal of Mindfulness-Based Stress Reduction (MBSR). This method was developed by Kabat-Zinn [10] in the scope of preventive and behavioral medicine at the Stress Reduction Clinic and the Center for Mindfulness in Medicine, Health Care, and Society at the University of Massachussets. This author designed an eight-week program with the main goal of becoming aware of one’s own life experience, going from experiencing a life based on “automaticity” to fuller, more conscious living based on enjoyment of daily tasks. Its premise is that mind and body form an insoluble connection, and a condition for being happy is knowing how the body expresses itself to control the mind. Mindfulness Based Cognitive Therapy (MBCT) emerged from the study by Kabat-Zinn and Santorelli [11] at the UMAS Massachusetts Medical Center in Boston. It began to be used with people with severe psychological or physical problems (major depression, etc.) and concentrates on the discourse of thoughts, combining MBSR techniques with Cognitive-Behavioral techniques. In addition, Dialectic Behavioral Therapy (DBT) [12] proposes acceptance of oneself to generate change as the purpose of therapy. It is recommended for Borderline Personality Disorder and trains in various personal, interpersonal, emotion regulation, tolerance to distress and full awareness skills. Similarly, the Acceptance and Commitment Therapy (ACT) was developed by Hayes et al. [13]. ACT means accept, choose and take action. Its main proposal is that by knowing their own cognitive processes, people can regulate their own behavior, since the relationships with events tend to be arbitrary. The four components of Acceptance and Commitment Therapy are: fusion, evaluation, avoidance and reason-giving (FEAR). Fusion highlights the predominance of what is verbalized about everything else. Evaluation checks whether what has been verbalized has been done. Avoidance involves trying not to have the experience. And reason-giving is a recurrent attempt to make sense of behavior considered inappropriate, but without solving the situation. ACT is intended to connect verbal discourse with life events, decrease experiential avoidance, accept one’s own intimacy and clarify personal values and live in harmony with them. Finally, the Relational Therapy Brief theory by Safron and Muran [14] considers reality to be constructed through relationships with others. Therapeutic intervention attempts to break with the rigid conception of what one is and try to go with the flow or be, by discovering or reflecting on past experiences, and further by different ways of experiencing new ones that break with the relational schemas constructed. It works on the here and now of the therapeutic alliance and is intended to place attention on the present, by practicing full attention.

These therapies originate from a positive focus on health, understood as the capacity for adaptation and self-management of social, physical and emotional challenges [15]. They include mindfulness, which involves becoming aware of and paying attention to the purpose of the present moment, nonjudgmentally, accepting things the way they are [16]. Training in them improves the scope and use of coping skills based on self-observation and attention [17]. It is a means of coping with stressful everyday life events, attempting to avoid the mind’s tendency to continual judgment and promoting a more reflexive, less reactive and impulsive response to internal and external stimuli [18,19]. Some authors have found that MBIs are also useful for coping with burnout [20], which can have serious repercussions on the psychological health of workers [21], and in which emotional intelligence also has a relevant role in prevention [22]. In the scope of family caregivers of patients with dementia, mindfulness, along with other psychological techniques, has been associated with a variety of benefits for psychophysical health [23]. The first hypothesis of this study emerges from this, expecting that after MBI intervention, family caregivers of dementia patients will have positive results and less depression. 

### 1.1. Mindfulness-Based Intervention and Family Caregivers of Persons with Dementia

The World Health Organization (WHO) defines dementia as a chronic or progressive syndrome characterized by deterioration of the ability to process thought, affecting cognitive function, and in particular, language, thinking, orientation, memory, calculation, comprehension and judgement [24]. It can be caused by brain injuries, its symptoms appearing from 65 years of age on, and Alzheimer’s Disease (AD) is one of its most frequent forms [25]. Due to the growing elderly population in today’s society, the incidence of dementia patients is expected to increase in the coming years, which will mean deployment of healthcare, social resources, and so forth, for them. 

Dementia not only affects the person who suffers from it, but also has repercussions on their caregivers [26]. Some authors have suggested that the life of an Alzheimer’s patient caregiver undergoes a radical change due to patient demands implicit in this type of disease [27,28]. Furthermore, progress of society in recent years toward full equality of rights and obligations of men and women [29] means caretaker overburden is indifferent of gender [30]. Therefore, caregivers, and in particular caregivers of dementia patients, constitute a high-risk group for mindfulness problems [31] and symptoms of stress and depression [32], as well as more use of drugs and hospitalization than those who are not caregivers [33]. The second hypothesis of this study, which expected caretakers who participated in mindfulness-based intervention to have lower levels of anxiety, stress and over burden, was proposed from this perspective. Caretaker overburden, understood as fatigue and exhaustion, can affect mood [34]. Based on this premise and to continue studying the subject in greater depth, the third hypothesis posed was that mindfulness-based intervention would generate higher levels of caregiver sleep quality.

### 1.2. The Present Study

Intervention programs must be supported by a synthesis of empirical evidence in which the heterogeneity of the different studies shows their true efficacy. Meta-analytical studies are designed to overcome the deficiencies and contradictions found in the literature by analyzing available empirical evidence [35]. This meta-analytical review focuses on the efficacy of full awareness-based interventions on psychological variables in the caregivers of dementia patients. Therefore, the main objective of this study was to analyze the efficacy of mindfulness-based intervention for psychological variables of family caregivers of persons with dementia. This general objective was broken down into the following specific objectives: (a) Study the influence of MBI on depression levels; (b) analyze anxiety, perceived stress and overburden levels of caregivers after MBI; and (c) find the effect of MBI on sleep quality of caretakers of dementia patients.

Based on previous empirical evidence, the following research hypotheses were posed:

**Hypothesis** **1:** Programs based on full awareness have positive results in lowering depression in caretakers of dementia patients;

**Hypothesis** **2:** MBI positively influences anxiety, perceived stress and caregiver burden;

**Hypothesis** **3:** IBM positively influences sleep quality, improving the personal wellbeing of caregivers.

## 2. Materials and Methods 

### 2.1. Procedure

An exhaustive databases search was made following the PRISMA recommendations by Moher, el al. and the PRISMA Group [36]. The databases queried were PubMed, Web of Science and PsycINFO. The Boolean operators AND and OR were used as the search strategies. First a search was made using the following descriptors: “Caregivers” AND “Dementia” OR “Alzheimer” AND “Mindfulness”. Then a second search was made adding one more descriptor: “Caregivers” AND “Dementia” OR “Alzheimer” AND “Mindfulness” AND Cognitive Impairment. The filter applied in databases was document type (article), with which a total of 282 articles were found for reading and reviewing. The search period included studies published from 1 January 2000 to 30 September 2019. Articles dealing with mindfulness and family caregivers of persons with AD were analyzed during the month of October.

### 2.2. Inclusion and Exclusion Criteria

Articles which met the following criteria were included: (1) Studies published from 2000 to 2019; (2) in English and Spanish; (3) that evaluated the relationship between mindfulness and psychological parameters, such as depression, anxiety, perceived stress, caregiver burden and sleep quality of caregivers of dementia patients; (4) were on intervention and provided numerical data necessary for a meta-analysis; (5) with access to the complete text; and (6) peer-review studies.

The exclusion criteria set were: (1) Duplicate studies; (2) No quantitative analysis of data; (3) published in a language other than English or Spanish; (4) related to other matters concerning Alzheimer’s or caregivers (any study not about mindfulness-based intervention); (5) did not show means (*M*) or standard deviations (*SD*) for pretest/posttest intervention in the Results section.

### 2.3. Data Extraction

First, a data extraction formula was designed that included information on the preestablished inclusion criteria (authors and year of publication, design, sample, variables evaluated, place where the study was performed and most characteristic results). 

Articles were selected after first reviewing the title and abstract for application of the first exclusion criterion. Then the complete text of the article was read thoroughly by three team members to be able to apply the rest of the conceptual and methodological criteria established. 

### 2.4. Risk of Bias

The Cochrane Collaborations evaluation tool for assessing risk of bias [37] was used. Individual studies were scored for risk (1 low risk, 2 high risk and 3 unclear risk). Bias risk was independently evaluated by two researchers, and disagreements were resolved by consensus in a meeting with the senior researcher. The information extraction process ensured that the psychological parameters studied (depression, anxiety, perceived stress, caregiver burden and sleep quality) were present in the articles which were finally analyzed. Then the potential for publishing bias of the studies included was evaluated by inspecting the points on a Funnel plot.

### 2.5. Data Analysis: Meta-Analysis

The *Intervention Review* option was selected to test the effectiveness of the interventions, following a random effects model. The meta-analysis was performed with the Cochrane Review Manager statistical program (RevMan), version 5.3. (Cochrane, London, UK), to test for heterogeneity of the studies, effect size, data quality, etc. [38]. Interpretation of the effect estimates (SMD) was in line with Cohen’s guidelines [39], where 0.2 is a weak effect, 0.5 medium and 0.8 large. The effect size is considered favorable if the result shows improvement in intervention. Heterogeneity is considered high if *I² ≥* 75%, medium from 50 to 75% and low when the *I²* ≤ 25% [34].

## 3. Results

**Figure 1:**
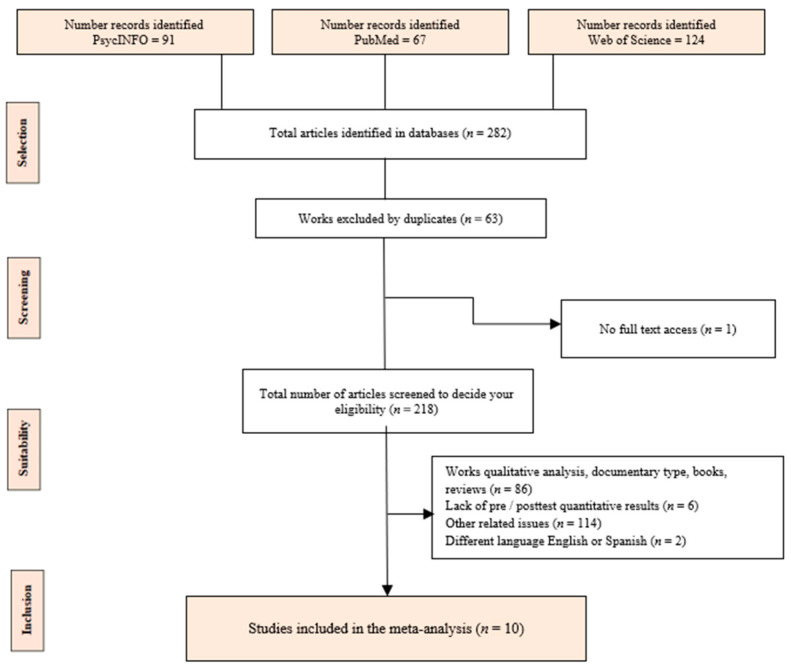
Flow diagram showing article selection steps.

Figure 1 shows the study selection process (flowchart). A total of 282 studies were identified, of which 63 duplicates were eliminated, leaving 206 studies to be reviewed. Later, another 194 articles were eliminated for various reasons as observed in Figure 1. Finally, a total of ten studies were included in the systematic review and meta-analysis.

The studies carried out mindfulness-based interventions and evaluated their efficacy by comparisons extracted in the pretest-posttest results. As shown in Table 1, four studies were uncontrolled trials with one group, four were uncontrolled trials with two groups, one was an uncontrolled trial with three groups, one uncontrolled trial had four groups, one study was a two-group controlled trial and one was a one-group randomized controlled trial. 

The sample size varied from nine to 35 participants. Most of them were women caregivers of persons with dementia. The mean sample age varied from 46, specifically, in the study by Franco et al. [30] and 70 in the study done by Berk et al. [15]. The time employed in the mindfulness-based interventions varied from one study to another. The shortest intervention time, which took an average of two hours a week for four weeks, was in the study by Hoppes et al. [16]. The intervention carried out by Whitebird et al. [17] was the longest, with eight two-hour sessions per week for six months. An exception was the study by Waelde and Gallagher-Thompson [23], which lasted two years, but in this case, after appropriate instruction had been given each participant, the sessions were performed individually at home for thirty minutes a day. 

To extract the information, the studies were coded as follows: (1) Authors and year of publication, (2) study design, (3) country, (4) variables evaluated, (5) sample, (6) duration, and (7) most relevant results after intervention (Table 1). The results concerning mindfulness and the variables evaluated in the studies selected for systematic review and meta-analysis are presented below. The total number of caregivers of persons with AD in all of the studies came to 161.

### 3.1. Summary of Results: Meta-Analysis

Six studies were excluded because the results section did not show the quantitative data necessary for the meta-analysis. Thus, the meta-analysis of a total of eleven studies was performed for the following variables: depression, anxiety, perceived stress, caregiver burden and sleep quality.

### 3.2. Result Variables for Depression, Anxiety, Perceived Stress, Caregiver Burden and Sleep Quality

**Figure 2:**
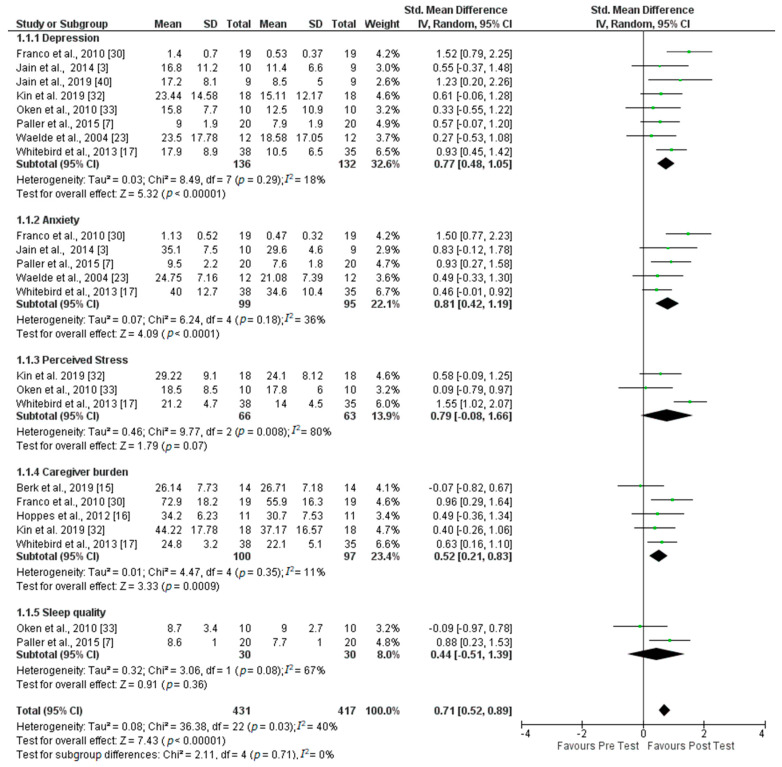
Forest plot of the effect size of mindfulness intervention on psychological variables Note: Tau² = estimate of variance of effect sizes; Chi² = existence of heterogeneity; df = global effect size indicator on the results of a meta-analysis; *p* = statistically significant heterogeneity; green dots = effect size of each study; black diamond in each variable = effect size of the set of studies; final black diamond = size of subgroup effect.

Ten studies with a total of 157 participants in the postintervention were selected. Figure 2 shows the results or effect sizes (magnitude of the relationship found in each study for each of the psychological variables entered in the analysis) of each of the comparisons, as well as the overall effect size of all the comparisons, as observed in the Forest plot (graphical display of estimated results in the studies along with overall results). The Forest plot specifically shows the central vertical axis corresponding to the null effect size, and all the effect sizes to the right of the axis show a favorable effect after intervention. 

Several different instruments were used for evaluation of the variables. For depression and anxiety, Franco et al. [30] used the SCL-90-R20 questionnaire. Other studies used the Quick Inventory of Depression Symptoms—Self Report (QIDS) to measure depression, the Center of Epidemiological Studies of Depression Scale (CES-D) or the Geriatric Depression Scale (GDS). Anxiety was evaluated using the Zung Anxiety Scale (ZAS), the Beck Anxiety Inventory (BAI) and the State-Trait Anxiety Inventory (STAI). However, to measure perceived stress, the studies all used the same instrument, the Perceived Stress Scale (PSS). To measure caregiver burden, the following instruments were used: The Self-Perceived Pressure from Informal Care (SPPIC), the Zarit Burden Scale (ZBI) and the Montgomery Borgatta Caregiver Burden Scale. Finally, for evaluation of sleep quality, they used the Pittsburgh Sleep Quality Index and Pittsburg Sleep Quality Inventory (PSQI).

The heterogeneity (*I*^2^), that is variability between study results, is given below for each of the comparisons, as well as the variance in effect sizes, using the Tau squared (Tau^2^), which estimates the variance in effect sizes, such that the largest absolute values indicate the strongest relationships. As shown in Figure 2, the results reflect low heterogeneity in the studies analyzing the effect of mindfulness intervention on depression (*I*^2^ = 18%), where the number of studies was *k* = 8, anxiety (*I*^2^ = 36%; *k* = 5) and caregiver burden (*I*^2^ = 11%; *k* = 5), moderate in the case of the effect of mindfulness intervention on sleep quality (*I*^2^ = 67%; *k* = 2) of the caregiver of dementia patients and high for the effect of mindfulness on perceived stress (*I*^2^ = 80%; *k* = 3). 

Overall heterogeneity was moderate (*I*^2^ = 40%). In almost all cases, after intervention, the direction of the effect size was considered favorable, except for the study by Berk et al. [10] on caregiver burden, which had a negative effect, as it did in the intervention by Oken et al. [33] on sleep quality. The intervention which showed the least effect on each variable would correspond to the one in which the green point was farthest to the right of the black diamond. For example, for depression, the study which showed the least effect was the one by Waelde and Gallagher-Thompson [23] on anxiety, the intervention by Whitebird et al. [17] and for perceived stress, the study by Oken et al. [33]. For caregiver burden, it was the study by Berk et al. [15], and, finally, for the sleep quality variable, it was the work by Oken et al. [33]. However, the results of the meta-analysis by subgroup showed that the interventions had a significant (*p* ≤ 0.00001) effect, with an SMD at a Confidence Interval (CI) of 95% = 0.71 (0.52, 0.89).

### 3.3. Risk of Bias in Variables

**Figure 3:**
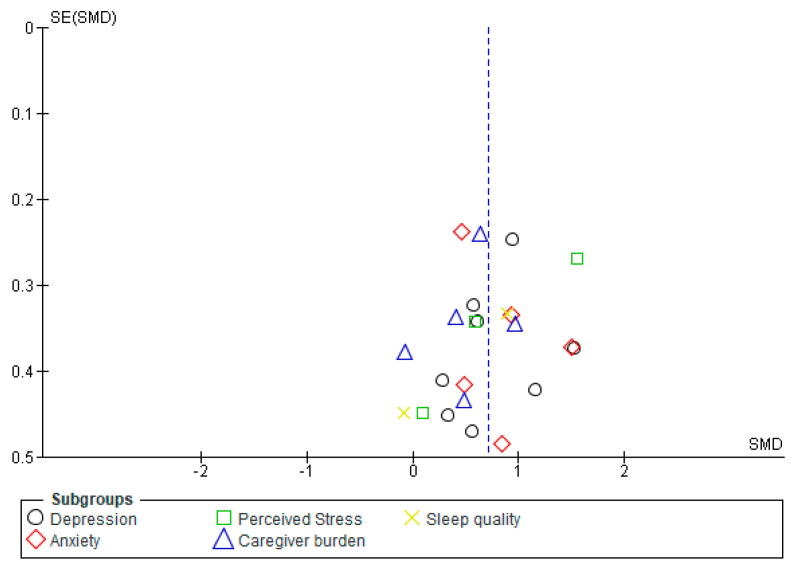
General risk of bias.

Once the studies in the meta-analysis had been selected, the reliability of the results was evaluated by risk of bias assessment. The risk of bias, according to the Cochrane risk of bias assessment tool, was not high as seen in Figure 3. The Funnel plot (graph verifying the existence of bias in publications) is shown below with the results for risk of bias in the psychological parameters studied. Inspection of the point distribution in the figure shows how each of the variables (depression, anxiety, perceived stress, caregiver burden and sleep quality) has a low risk of bias. 

## 4. Discussion

The objective of this study consisted of analyzing the efficacy of mindfulness-based interventions on psychological variables of family caregivers of persons with dementia. The studies focused on different aspects. Franco et al. [30] performed an intervention for caregivers of persons with AD in which they evaluated psychological distress and caregiver overburden. After intervention, they found positive results, and concluded that the caregivers showed a reduction in overburden, coinciding with the results found by Hoppes et al. [16]. These findings were probably due to MBI being able to significantly reduce anxiety, depression and psychological distress [5]. 

In another of the analyzed studies, Jain et al. [3] showed that in the stage prior to mourning, caregivers are exposed to continual stressors which can cause symptoms of depression, anxiety, stress and overburden, in addition to less hope [15]. On the contrary, application of MBI has been related to an increase in self-efficacy [33], making it possible to fight stress [17] and improve the caregiver’s quality of life [7].

Kin et al. [32] found that after mindfulness intervention, family caregivers of persons with AD were able to manage daily challenges, becoming more tolerant and accepting situations more optimistically. In this respect, several authors thought yoga and meditation in mindfulness increased perceived self-efficacy, quality of life, attention, self-compassion and vitality [23]. Considering that one of the main goals of MBI is helping people (in this case caregivers) to learn and apply awareness exercises in their daily lives to help them understand and accept factors affecting them, adherence to this practice in the home may improve if the professionals provide caregivers with the corresponding previous instruction, and then maintain constant follow-up of their participants [23,31]. 

This meta-analysis confirmed the efficacy of different mindfulness-based interventions, as the majority of the studies were favorable after their application on different variables: depression, perceived stress, caregiver burden and sleep quality, and therefore the three hypotheses posed in our study may be accepted. It should be emphasized in turn, that based on the meta-analytical data found, the time of intervention did not seem to influence the program results, as interventions lasting a shorter time [7] had a stronger effect than others with a longer intervention time [23]. This could be due to the frequency of intervention program participation. 

Bias was low in the various domains. Our results showed that mindfulness-based intervention programs contributed to improvement in health and wellbeing of family caregivers of persons with dementia [26]. Total heterogeneity found in the meta-analytical results was moderate. However, when heterogeneity was higher, as in perceived stress and sleep quality, this could have been due to the differences in the methodological focus employed by the studies in the meta-analysis, or else to the characteristics of the mindfulness interventions, or others. 

Summarizing, the moderate effect may show that mindfulness-based practice should be completed with other coping strategies or that other variables, such as personal variables, could be mediating in the efficacy of mindfulness-based intervention, which would then be more effective in certain cases depending on the variables. Among the main goals of mindfulness is to improve a person’s wellbeing. Intervention programs based on full attention also improve the person’s perception of health [32]. Therefore, its implementation brings about favorable changes in negative symptoms present in the caregiver of dementia patients, thereby improving their quality of life [40].

### 4.1. Limitations

However, one of the main limitations of this study was that the sample sizes did not vary much from one study to another. Another of the limitations is related to the search strategy which focused on the Web of Science, PsycINFO and PubMed databases, so that studies published in other resources may have been omitted involuntarily. Some of the studies found were not included in the meta-analysis because of the lack of quantitative data in the results section. Finally, another of the limitations is the variety in the instruments used to measure the variables, which may have affected the heterogeneity found in the study.

### 4.2. Recommendations for Future Research

As a future line of research, new studies should incorporate longitudinal designs, since most of the studies on this subject that we were able to query employed cross-sectional designs. New comparisons could thus be made and new conclusions might be arrived at that could establish causal relationships between the effects of mindfulness-based intervention and their psychological benefits in this context. We also suggest that new studies make a quantitative analysis of data, in addition to qualitative, to enable meta-analytical studies to contribute to progress in research on the subject. 

### 4.3. Practical Applications of Results

This study could show the implications of Mindfulness for developing a positive psychological state in caregivers of dementia patients. Given today’s proximity, availability and ease of access to mindfulness-based interventions, it is recommended that they be put into practice for improving health, wellbeing and quality of life of family caregivers of persons with this type of illness. 

## 5. Conclusions

After the review of the studies and their meta-analysis, it may be concluded that mindfulness-based intervention has a positive effect on various psychological aspects. Based on these results, the design of intervention techniques and programs involving extended practice of mindfulness is relevant, since its prolonged practice improves intervention efficacy. 

The meta-analytical data found suggest that the time the intervention lasts does not seem to influence the results of the program. Therefore, participation for a minimum time could have great potential for the wellbeing of the caregivers, as long as the frequency of attendance is adequate.

In conclusion, MBI is an effective psychological resource for family caregivers of persons with dementia, as the results showed a favorable effect on the variables analyzed, improving quality of life and psychophysical wellbeing of caregivers of dementia patients, as stress, anxiety, depression and overburden were reduced, and sleep quality improved after intervention. 

## Figures and Tables

**Table 1:** Studies dealing with the relationship between mindfulness and caregivers.

Authors, (Publication Year) [Reference]	Study Design	Place	Variables Evaluated	*N*	Duration	Most Characteristic Results
Berk, Warmenhoven, Stiekema, van Oorsuow, van Os, de Vugt, and van Boxtel, 2019 [15]	Uncontrolled trial, 1 group	Mexico	Psychological distress, mindfulness, self-compassion, mental health, worry, caregiver burden and self-esteem	14	8 weeks (2.5 h/week)	Caregivers show less psychological distress and increased attention to quality of life
Jain, Nazarian and Lavretsky, 2014 [3]	Uncontrolled trial, 1 group	Germany	Depression, anxiety, quality of life, enjoyment and satisfaction, insomnia and mindfulness	10	8 weeks (3 weekly sessions of 1 h–15 min sessions)	Pain, depression and mindfulness were interrelated, but their neural mechanisms did not overlap, at least partly
Whitebird, Kreitzer, Crain, Lewis, Hanson, and Enstad, 2013 [17]	Uncontrolled trial, 2 groups	New York	Mindfulness, stress, depression, anxiety and caregiver burden	38	2 months (8 weekly sessions of 2.5 h)	Caregivers showed lower stress and depression
Hoppes, Bryce, Helman and Finlay, 2012 [16]	Uncontrolled trial, 2 groups	UK	Burden, hope, optimism and mindfulness	11	4 weeks (1 h/week)	Caregivers showed greater acceptance, presence, peace and hope, and decreased caregiver burden
Oken, Fonareva, Haas, and Wahbeh, 2010 [33]	Uncontrolled trial, 3 groups	Portland	Mindfulness, cognitive means, physiological means, expectations and sampling based on experience	10	7 weeks (90 min/week)	Caregivers showed higher self-efficacy and cognitive means
Waelde and Gallagher-Thompson, 2004 [23]	Uncontrolled trial, 1 group	San Francisco	Depression, anxiety, self-efficacy and mindfulness, caregiver burden and subjective improvement of perceived self-efficacy	12	2 years (30 min/week)	Caregivers revealed significant reductions in depression and anxiety, and improvement in perceived self-efficacy
Franco, Sola, and Justo, 2010 [30]	Controlled trial, 2 groups	Spain	Caregiver load, self-efficacy, somatization, interpersonal sensitivity, depression, anxiety, hostility, phobic anxiety, paranoid ideation distress	19	10 weeks (10 sessions of 2 h)	Caregivers showed less psychological distress in general, and less overburden
Jain, Connolly, Moore, Leuchter, Abrams, Ben-Yelles, Chan, Ramirez, Lavretsky and Lacobini, 2019 [40]	Uncontrolled trial, 1 group	Los Angeles	Mourning, depression and mindfulness	9	4 weeks (2 h/week)	Caregivers showed lower levels of depression, sorrow for loss, and increased mindfulness
Kin, Wa, and Tong (2019) [32]	One-group randomized controlled trial	China	Stress, caregiver burden, depression, anxiety, resilience, quality of life and mindfulness	18	10 weeks (7 sessions of 2 h)	Caregivers showed reduction in stress levels and improved psychological wellbeing
Paller, Creery, Florczak, Weintraub, Mesulam, Reber, Kiragu, Rooks, Safron, Morhardt, and O’Hara, 2015 [7]	Uncontrolled trial, 4 groups	Evanston	Quality of life, depression, anxiety, subjective sleep quality, visual attention and socioemotional and physical health	20	8 weeks (8 weekly sessions of 1 h–30 min sessions)	Caregivers showed reduction in anxiety levels, lighter symptoms of distress and better sleep quality and quality of life
